# A global dataset for prevalence of *Salmonella* Gallinarum between 1945 and 2021

**DOI:** 10.1038/s41597-022-01605-x

**Published:** 2022-08-13

**Authors:** Xiao Zhou, Xiamei Kang, Kun Zhou, Min Yue

**Affiliations:** 1 Institute of Preventive Veterinary Sciences & Department of Veterinary Medicine, Zhejiang University College of Animal Sciences, Hangzhou, 310058 China; 2 Hainan Institute of Zhejiang University, Sanya, 572025 China; 3 State Key Laboratory for Diagnosis and Treatment of Infectious Diseases, National Clinical Research Center for Infectious Diseases, National Medical Center for Infectious Diseases, The First Affiliated Hospital, College of Medicine, Zhejiang University, Hangzhou, 310003 China; 4 Zhejiang Provincial Key Laboratory of Preventive Veterinary Medicine, Hangzhou, 310058 China

**Keywords:** Infectious-disease epidemiology, Agriculture

## Abstract

Pullorum disease and fowl typhoid are among the most significant poultry diseases worldwide. However, the global burden of these diseases remains unknown. Most importantly, the parameters contributing to the prevalence of *Salmonella* Gallinarum variants are not well documented. Therefore, in this study, we present a systematic review and meta-analysis of the global prevalence of *Salmonella* Gallinarum during 1945–2021. In total, 201 studies were identified for qualitative analysis (>900 million samples). The meta-analysis was subjected to over 183 screened studies. The global prevalence of *S*. Gallinarum (percentage of positive samples in total samples) was 8.54% (95% CI: 8.43–8.65) and showed a V-shaped recovery over time. Pullorum disease is most common in Asia, particularly in eastern China. Further investigations on chicken origin samples revealed significant differences in *S*. Gallinarum prevalence by gender, breed, raising mode, economic use, and growth stage, indicating a critical role of vertical transmission. Together, this study offered an updated, evidence-based dataset and knowledge regarding *S*. Gallinarum epidemics, which might significantly impact decision-making policy with targeted interventions.

## Abstract (summary)

**Table:** 

Measurement(s)	*S*. Gallinarum positive samples
Technology Type(s)	Serological test • Molecular method (PCR) • Plate culture and identification • Biochemical test
Factor Type(s)	biovar • time • geographic sampling site • reservoir of bacteria • chicken breed • raising mode • economic use • growth stage • gender
Sample Characteristic - Organism	Animal
Sample Characteristic - Location	world

## Background & Summary

The global demand for meat has been continuously expanding for several reasons, including a growing population, increased income levels, and accelerated urbanization^1–4^. In 1961, poultry meat accounted for only 12% of global meat production, which increased to 36% by 2016. Moreover, global demand for poultry is projected to increase by 121% by 2050 compared with 2007, becoming the most significant increase of any other meat type^5^. In 2021, the United States had the highest volume of chicken meat production among all countries, producing about 20.3 million tons of chicken meat. During the same period, China ranked second with 15 million tons, while Brazil ranked third with 14.2 million tons of production^6^. Chicken becomes the fastest growth in livestock sectors due to its high-quality protein with low price, short reproductive cycles and efficiencies in converting feed into meat compared to other meats^7–10^. Especially in China, African Swine Fever (ASF) epidemics since August 2018 has pushed consumers towards chicken, replacing pork^11–13^. The poultry sector in China has experienced vigorous growth over the past two decades. However, sustainable development in the global poultry industry has several key limitations, including the emergence and re-emergence of infectious diseases in poultry industry.

Pullorum disease and fowl typhoid, caused by *Salmonella enterica* subspecies *enterica* serovar Gallinarum biovars Pullorum (bvSP) and Gallinarum (bvSG), respectively, are among the most important diseases listed by World Organization for Animal Health (OIE) and have resulted in considerable economic losses to poultry industry over the world^14–18^. bvSP and bvSG have been under complete control or eradication programs for decades in several industrialized countries^14,19–21^. However, they are still common in many regions, particularly in developing countries^14,19,21–23^. These two biovars are highly adapted to avian species with limited potential for food poisoning in humans and can be spread by both horizontal and vertical transmissions^24–28^. The severity of both diseases varies with several parameters, including the bird’s species, breed, gender, age, flock nutrition, and management, but mortality rates can approach 100% in highly susceptible birds. Pullorum disease is severe septicemia mainly in young birds, while fowl typhoid affects birds of all ages but is more often observed in growing and adult birds with clinical signs such as anorexia, diarrhoea, dehydration and ultimate death outcome^29^. In certain conditions, these two causative agents can also colonize and persist in birds for long periods and may be carried without any clinical disease symptoms or lead to decreased egg production and poor hatchability, which increase difficulties in disease control measures and significantly reduce the production and quality of poultry meat and eggs^30^. However, compared with other *Salmonella* serovars, there are few studies on *Salmonella* Gallinarum (bvSP and bvSG), resulting in inadequate knowledge of these two organisms. Moreover, there is a lack of systematic and comprehensive understanding of the burden of pullorum disease and fowl typhoid over the world, particularly in China, which serves as the second-largest poultry producer but has been plagued by the diseases for a long time.

We collected the up-to-date results from available studies involving the prevalence of *S*. Gallinarum published to date and conducted a systematic review and meta-analysis of the epidemiological parameters of *Salmonella* Gallinarum in China and globally, respectively. We aimed to provide updated and complete information on *S*. Gallinarum globally and offered evidence-based factors for targeted control measures.

## Methods

A meta-analysis-based systematic review of the global prevalence of *Salmonella* Gallinarum (1945–2021) was conducted according to the Preferred Reporting Items for Systematic reviews and Meta-Analyses (PRISMA) statement^31^ guidelines and the following methods.

### Search strategy in the databases

The search strategy was followed to ensure that the published literature around the globe before 2021 related to the prevalence of *Salmonella enterica* serovar Gallinarum should be collected and included in this systematic review for further processing and screening.

The literature search was conducted in both Chinese and English literature databases. The Chinese literature was searched in the China National Knowledge Infrastructure (CNKI) (http://www.cnki.net/), and in English in the PubMed database (https://pubmed.ncbi.nlm.nih.gov/) using the “鸡白痢” or “鸡伤寒” “Pullorum” or “Gallinarum” keywords in the search string to find all the related studies to the prevalence of *Salmonella enterica* serovar Gallinarum, published before May 1, 2021. Non-Engliash and non-Chinese literatures were not included in this study.

### Inclusion and exclusion criteria

**Fig. 1:**
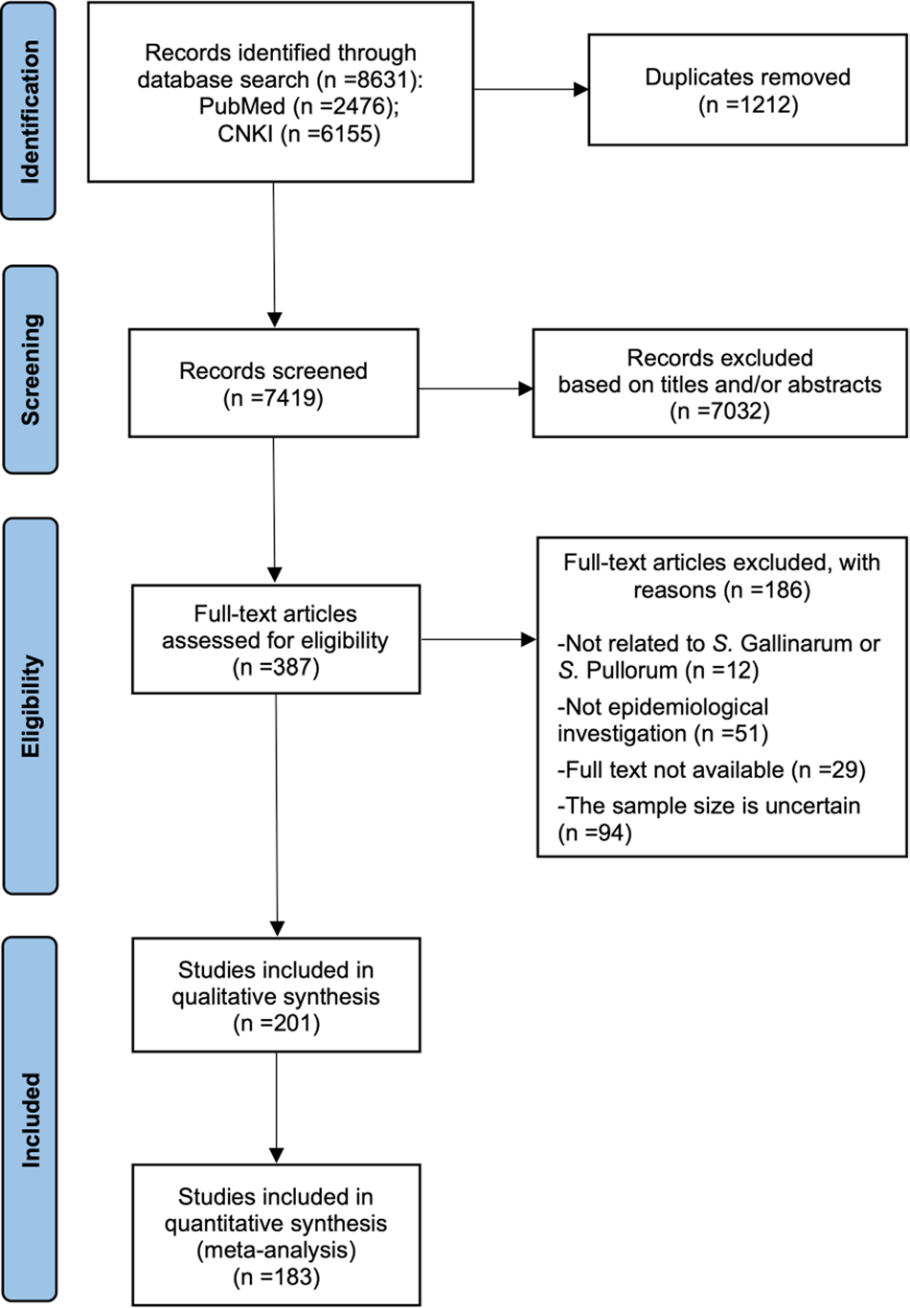
The flowchart for study selection.

**Fig. 2:**
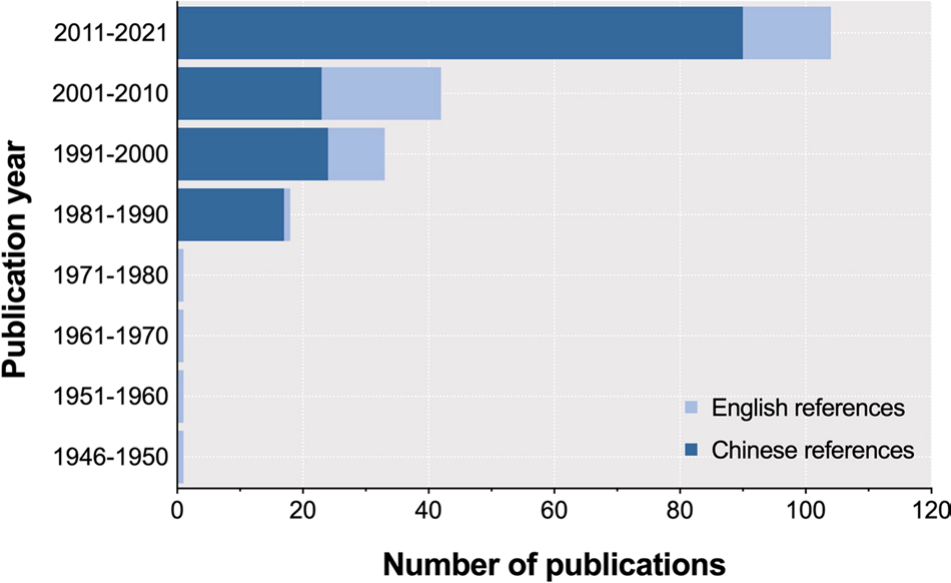
Number of Chinese and English publications recorded during 1945–2021.

The literature search and selection procedure is summarized in a flow diagram (Fig. 1). Initially, a total of 8,631 studies related to the *S*. Gallinarum (bvSP and bvSG) were published from 1945 to 2021, including 2,476 from PubMed and 6,155 from CNKI, were retrieved by the search strategy. And the collected studies were entered into EndNote to remove the 1,212 duplicate articles. The inclusion criteria for the remaining articles were set as they must include an epidemiological investigation of *S*. Gallinarum. 7,218 articles were excluded in this systemic review in which the abstract and full texts were unavailable, and the studies were found uncertain with the sample size. In total, 201 studies (47 English and 154 Chinese) were retained as per the criteria (Fig. 2).

### Data extraction

The key data extracted from the literature included the title of the research article, geographical location, type of the bacteria, sampling time, sample size, and sampling source. And the data undergo a number of strict validations to ensure the highest quality. A total of 520 records were included from PubMed Databse in English (95) and CNKI Database in Chinese (425). Characteristics of the included studies are shown in the **Figshare data**^32^.

### Sample description

The total sample size was over 900 million, among which 900,018,404 were tested for bvSG, 1,187,023 for bvSP, 286,282 for *S*. Gallinarum with unclear biotype. The samples were primarily collected from avians, mainly chicken. In contrast, few samples were collected from food, environment, arthropod, etc. In these samples, it was found that the rapid slide agglutination test was the most common diagnostic technique used for the detection of positive samples, followed by molecular tests, mainly polymerase chain reaction, bacteriological identification of the agent and biochemical tests.

### Geo-positioning

**Fig. 3:**
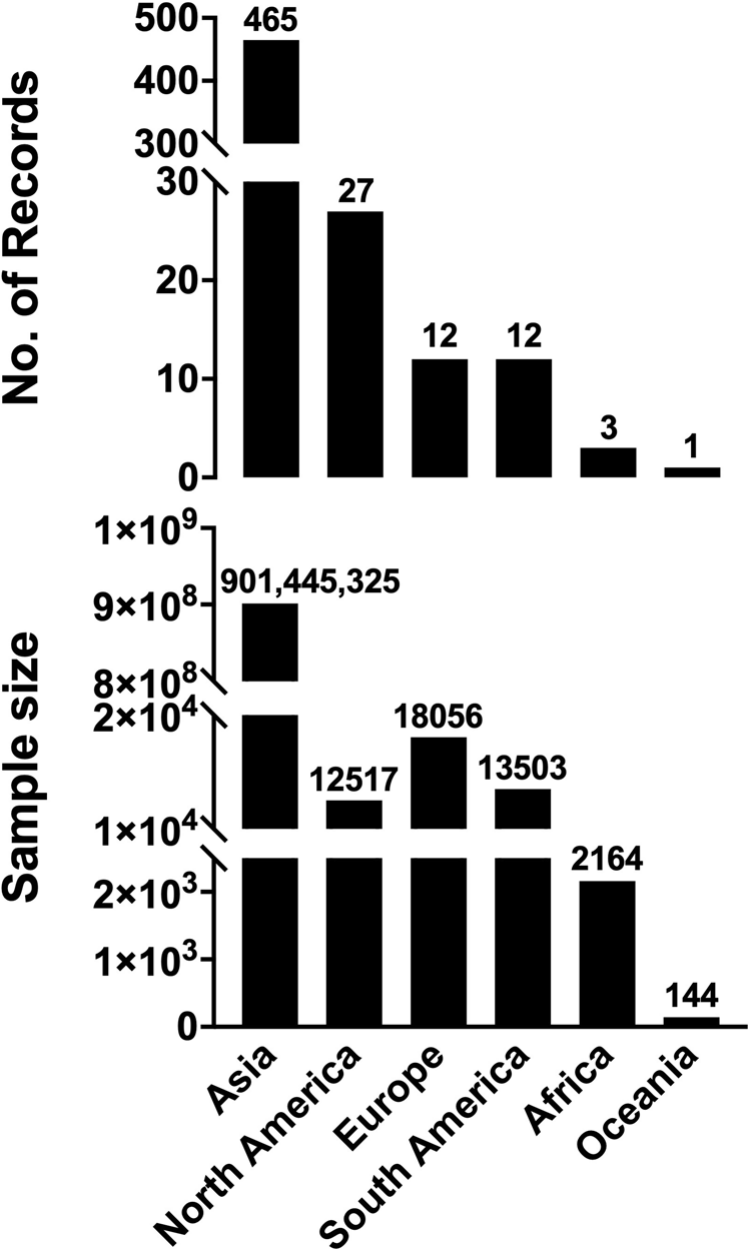
Distribution of sampling size and data records. Data used in this graph have been summarized in the **Figshare data**.

**Fig. 4:**
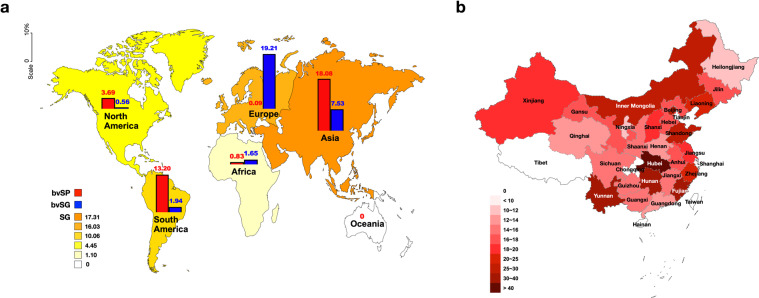
Descriptive analysis for the geographical distribution of *S*. Gallinarum. (**a**) Global geographical distribution of *S*. Gallinarum. (**b**) Geographical distribution of *S*. Gallinarum in China. Data used in this graph have been summarized in the **Figshare data**.

**Fig. 5:**
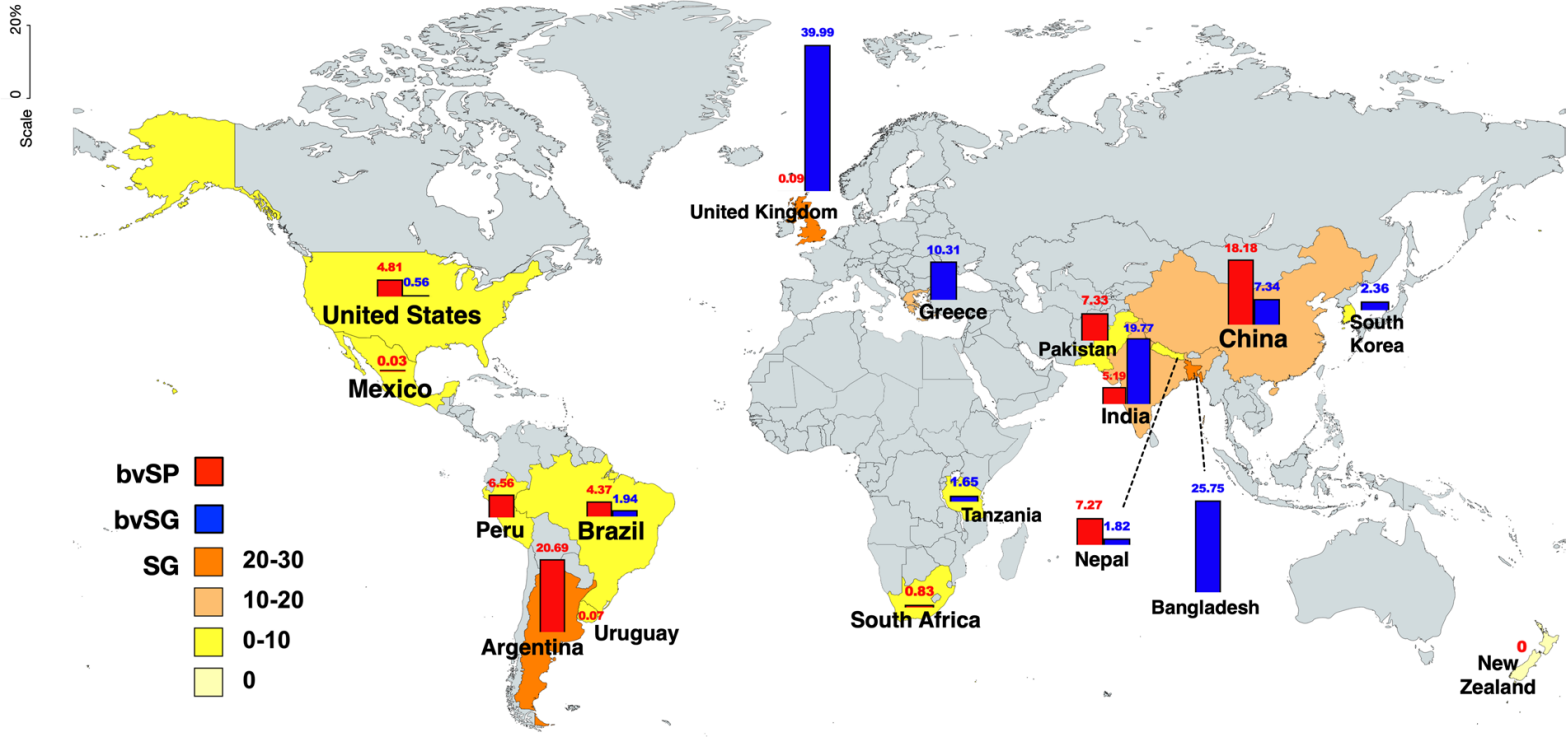
Distribution of *S*. Gallinarum in countries.

The geographical distribution of all the collected and screened studies reported that the total included studies in this systemic literature review were from 17 different countries on six continents. The total number of records in Asia was 465, with a sample size of 901,445,325. A total number of records in North America was 27, with a sample size of 12,517. A total number of records in Europe was 12, with a sample size of 18,056. A total number of records in South America was 12, with a sample size of 13,503. A total number of records in Africa was 3, with a sample size of 2,164. The number of record in Oceania was only 1 with a sample size of 144 (Fig. 3). The prevalence of *S*. Gallinarum (bvSP and bvSG) in 17 countries on six continents was investigated and analyzed. The results showed that all the continents observed positive prevalences of *S*. Gallinarum except Oceania. Whereas Asia had the highest prevalence at 17.31% and Europe at 16.3%. In Asia, the prevalence of bvSP was higher than bvSG, but in Europe, bvSG was observed to be two hundred times greater than bvSP. The prevalence of *S*. Gallinarum was higher (10.06%) than bvSP (13.20%) in South America. The prevalence of *S*. Gallinarum was relatively mild in North America (4.45%) than in Africa (1.10%) (Fig. 4a). Moreover, the prevalence of *S*. Gallinarum observed in 17 countries was analyzed, which reported that it was high in Bangladesh (25.75%), the U.K. (24.03%) and Argentina (20.69%), respectively. Whereas the prevalence of bvSP was highest in Argentina (20.69%) and China (18.18%). Prevalence of bvSG was highest in the U.K. (39.99%), Bangladesh (25.75%) and India (19.77%) (Fig. 5).

In China, literature (160), records (443) and sample sizes were significantly high compared to other countries. Therefore, comprehensive elaboration of the prevalence of *S*. Gallinarum was needed at the provincial level for in-depth understanding except for Tibet, Tianjin, Hainan, Hong Kong, Macao and Taiwan (Fig. 4b).

As per records, it was observed that the load of *S*. Gallinarum was extremely high in China. In addition, the largest number of records (n = 35) and samples (n = 660, 643) were from Heilongjiang, while only one record was extracted from Hubei and Hunan, respectively. In Fig. 4b, the prevalence of *S*. Gallinarum reported in all regions was positive. Nine regions were more than 20% and Hubei province had the highest rate of 75.07%. Most importantly, the prevalence of *S*. Gallinarum in Shanghai was the lowest at 7.92%.

### Meta-analysis

The data were further processed for meta-analysis under rigorous screening rules to obtain high-quality data for meta-analysis. The rigorous screening rules include: (1) sample size should not be less than *n* = 50; (2) sampling date should be clear; (3) sample source must be mentioned. In total, 183 studies (35 English and 148 Chinese) covering 468 records of high quality were screened for meta-analysis, and the characteristics of the examined studies are shown in the **Figshare data**^32^. The meta-analysis is conducted by using the binary random-effects model with a 95.0% confidence level using Open Meta-Analyst as previously described^33–35^. Furthermore, results were compiled in Microsoft Excel-16 and GraphPad Prism-9 and presented in graphs.

#### Meta-analysis for the spatial distribution of S. Gallinarum

**Fig. 6:**
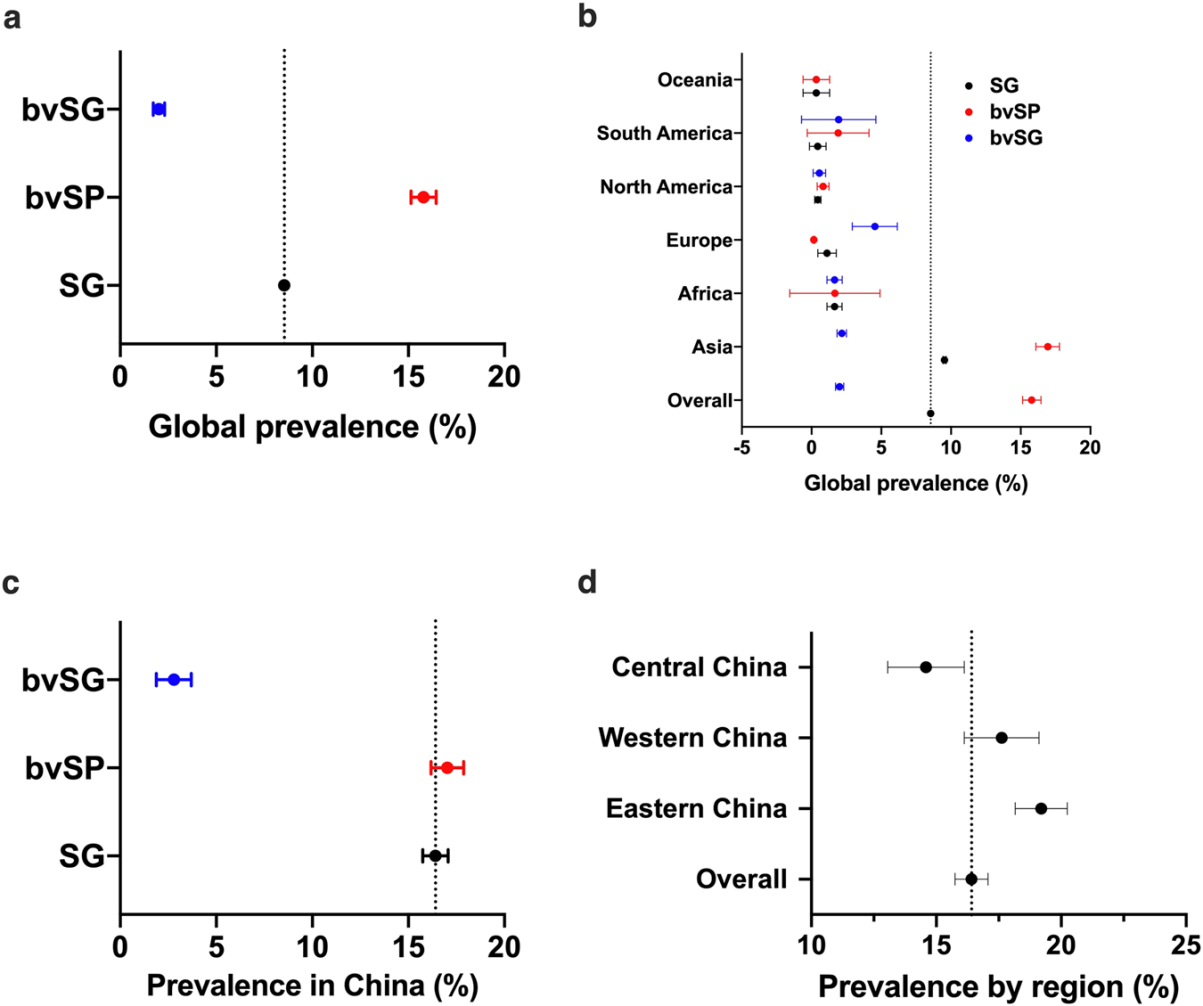
Meta-analysis for spatial distribution of *S*. Gallinarum. (**a**) Global prevalence of *S*. Gallinarum. (**b**) The prevalence of *S*. Gallinarum varied by continent. (**c**) The prevalence of *S*. Gallinarum in China. (**d**) The prevalence of *S*. Gallinarum varied by region in China. The broken vertical line indicates the pooled summary estimate of prevalence for the selected variable and unclassified data. Data used in this graph have been summarized in the **Figshare data**.

High-quality records (n = 468), which belong to 183 studies, were screened for evidence-based meta-analysis. The prevalence of *S*. Gallinarum in the globe from 1945 to 2021 was 8.54% (95% CI: 8.43–8.65), with a high global prevalence of bvSP at 15.79% (95% CI: 15.14–16.45) and a relatively low global prevalence of bvSG at 2.01% (95% CI: 1.72–2.31) (Fig. 6a).

In this study, a detailed analysis was conducted regarding the geography, time, and origin of the samples. The prevalence of *S*. Gallinarum varied across continents. The prevalence and records found higher in Asia (433 records, 9.53%, 95% CI: 9.42–9.65), higher than overall prevalence of *S*. Gallinarum and other continents, followed by Africa (2 records, 1.65%, 95% CI: 1.11–2.19), Europe (8 records, 1.11%, 95% CI: 0.45–1.77), North America (17 records, 0.45%, 95% CI: 0.23–0.67), South America (7 records, 0.44%, 95% CI: −0.15–1.04) and Oceania (1 record, 0.34%, 95% CI: −0.61–1.30). Interestingly, Europe had the lowest prevalence of bvSP (2 records, 0.16%, 95% CI: 0.10–0.23) but the highest of bvSG (6 records, 4.54%, 95% CI: 2.92–6.15). However, bvSP (328 records, 16.94%, 95% CI: 16.09–17.78) was the most prevalent in Asia, dominating the global prevalence (Fig. 6b).

**Fig. 7:**
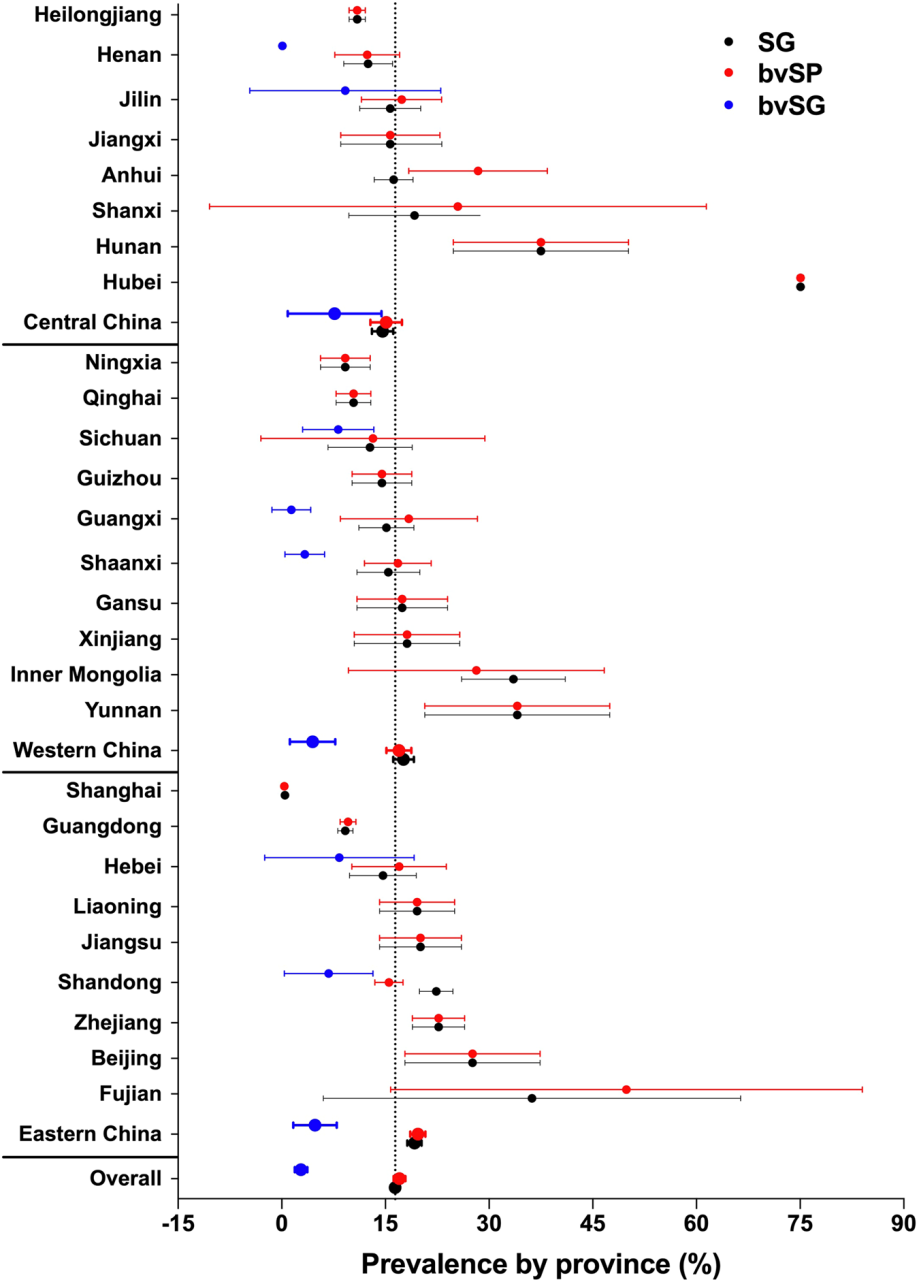
Distribution of S. Gallinarum in provinces. Provinces are sorted by region.

Whereas in China, the prevalence of *S*. Gallinarum was found to be the most serious in the world (411 records, 16.41%, 95% CI: 15.75–17.07), bvSP (325 records, 17.03%, 95% CI: 16.18–17.88) was six times more than bvSG (14 records, 2.79%, 95% CI: 1.88–3.7) in China (Fig. 6c). Moreover, in this study, the prevalence of *S*. Gallinarum was calculated region-wide, and the results showed that the prevalence was highest in eastern China (129 records, 19.20%, 95% CI: 18.16–20.24), followed by western China (126 records, 17.62%, 95% CI: 16.12–19.11), and relatively low in central China (112 records, 14.59%, 95% CI: 13.06–16.12) (Fig. 6d). The prevalence of *S*. Gallinarum, bvSP and bvSG in different regions and provinces of China are presented in detail in Fig. 7.

#### Meta-analysis for temporal distribution of S. Gallinarum

**Fig. 8:**
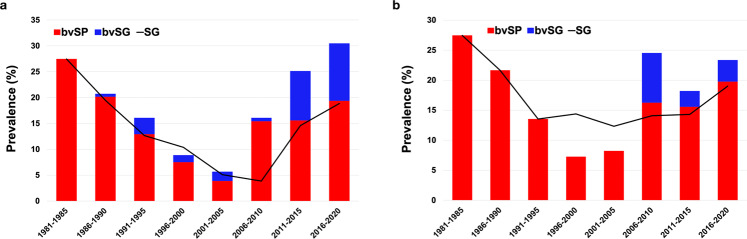
Meta-analysis for temporal distribution of *S*. Gallinarum between 1981 and 2020. (**a**) Global temporal distribution of *S*. Gallinarum. (**b**) Temporal distribution of *S*. Gallinarum in China. Data used in this graph have been summarized in the **Figshare data**.

The results showed that the global prevalence of *S*. Gallinarum was highest at the beginning (27.47%, 95% CI: 16.65–38.29) and decreased over time during 2006–2010, and then increased (18.87%) by 2020 (95% CI: 17.16–20.38). The prevalence of bvSP overtime was consistent with that of *S*. Gallinarum, which decreased gradually and reached the lowest value during 2001–2005 (3.89%, 95% CI: 3.20–4.58), whereafter increased to 19.38% (95% CI: 17.66–21.09) by 2020. The prevalence of bvSG remained low (0.56–3.17%) before 2010 but increased (9.54–11.11%) after 2011 (Fig. 8a).

**Fig. 9:**
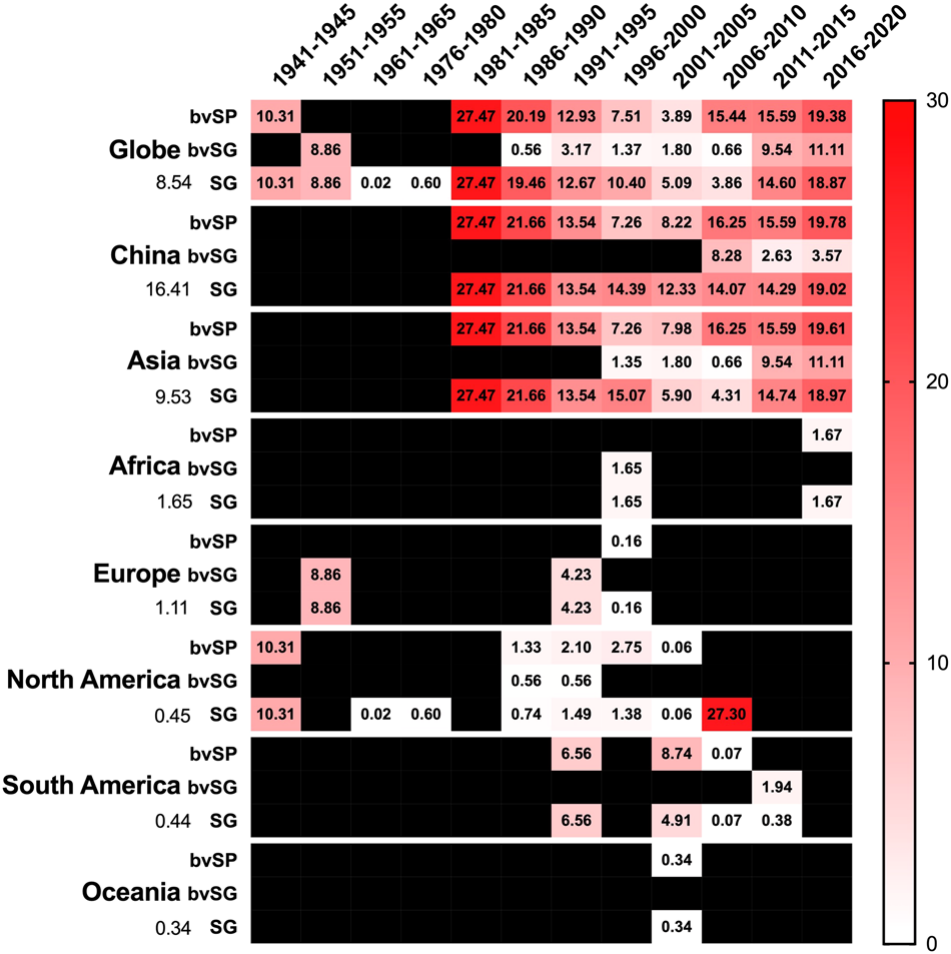
The spatiotemporal distribution of *S*. Gallinarum. The color of individual cells varies with the estimate of prevalence shown in the cells. Black cells mean no record. The numbers on the left represent the overall prevalence of *S*. Gallinarum for each region.

The earliest sampling time for records from China was 1983. These results showed that the prevalence of *S*. Gallinarum in China was severe in the early stage (21.66–27.47%), then gradually decreased and remained stable at 12.33–14.39% from 1991 to 2015, and increased in recent years (19.02%) during 2016–2020. The prevalence of bvSP also showed a V-shaped trend over time, reaching 27.47% (95% CI: 16.65–38.29) and 19.78% (95% CI: 18.05–21.52) in the early and late stages, respectively, and dropping to 7.26% (95% CI: 6.34–8.18) from 1996 to 2000. The prevalence of bvSG did not appear in China until 2006 (Fig. 8b). The prevalence of *S*. Gallinarum, bvSP and bvSG in different continents and China is presented numerically (Fig. 9**)**.

#### Meta-analysis for source distribution of *S*. Gallinarum

**Fig. 10:**
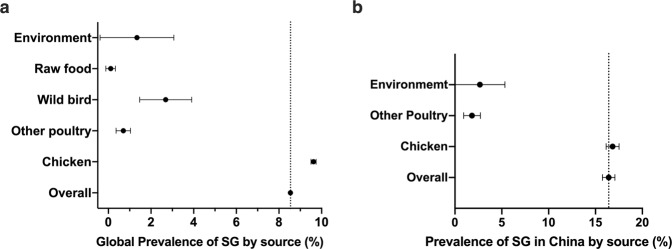
Meta-analysis for source distribution of *S*. Gallinarum. (**a**) Global source distribution of *S*. Gallinarum. (**b**) Source distribution of *S*. Gallinarum in China. Sources with records less than three are not shown. Data used in this graph have been summarized in the **Figshare data**.

**Fig. 11:**
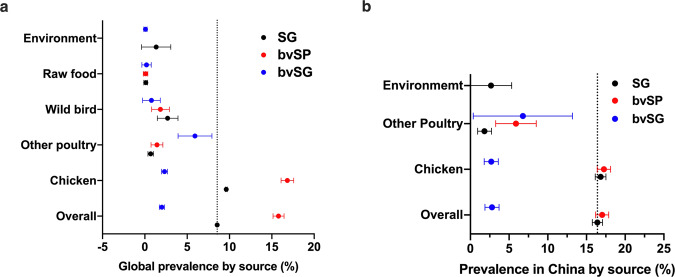
The source distribution of *S*. Gallinarum. (**a**,**b**) Sources with records less than three are not shown.

The number of records collected from the chicken source was 422, followed by other avian sources (n = 21) and wild birds (n = 12). Globally, the prevalence of *S*. Gallinarum detected in the chicken was much higher than in other reservoirs (9.62%, 95% CI: 9.50–9.74). The prevalence of bvSG was the highest in other poultry (5.92%, 95% CI: 3.93–7.92) (Figs. 10a, 11a).

In China, the prevalence records of *S*. Gallinarum from chicken sources, other avian sources, and the environment were 396, 13, and 2, respectively. The prevalence of SG from chicken in China was higher than that in the world (16.83%, 95% CI: 16.14–17.52) and more than six times higher than that of the other two reservoirs (Figs. 10b, 11b).

#### The prevalence of *S*. Gallinarum in chicken

**Fig. 12:**
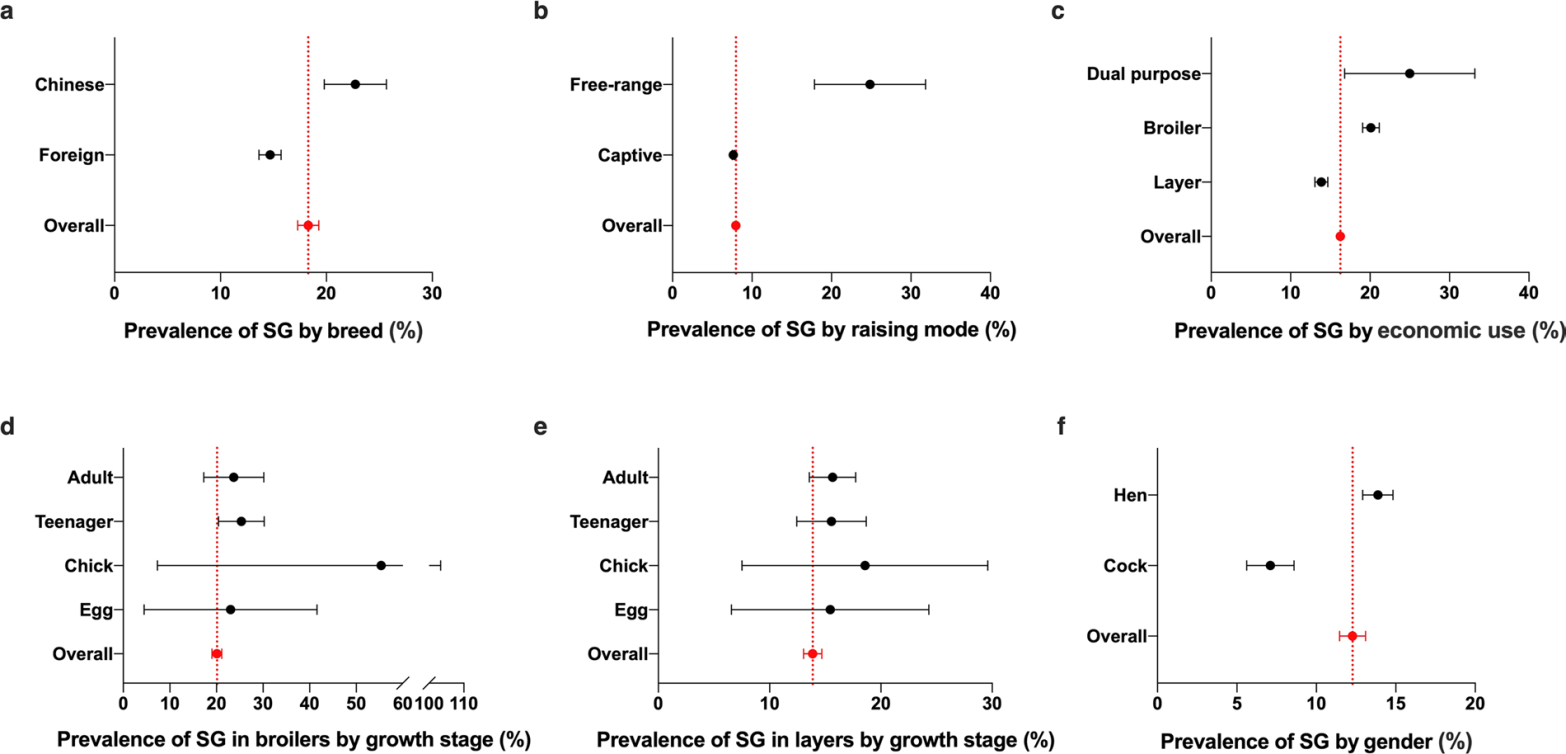
Subgroup meta-analysis for the prevalence of *S*. Gallinarum in chickens. (**a**) Breed distribution of *S*. Gallinarum in chickens. (**b**) Farming mode distribution of *S*. Gallinarum in chickens. (**c**) Economic use distribution of *S*. Gallinarum in chickens. (**d**) Growth stage distribution of *S*. Gallinarum in broilers. **(e)** Growth stage distribution of *S*. Gallinarum in layers. (**f**) Gender distribution of *S*. Gallinarum in chickens. Data used in this graph have been summarized in the **Figshare data**.

Meta-analysis was conducted for all the data of chicken origin on five variables, including gender, breed, raising mode, economic use and growth stage. The detailed results are presented (Fig. 12a–f).

To analyze the records accurately and comparatively, all the chicken breeds were classified into foreign and Chinese breeds. The prevalence of *S*. Gallinarum in chickens of Chinese breeds (98 records, 22.46%, 95% CI: 19.68–25.24) was higher than in foreign breeds (111 records, 13.90%, 95% CI: 12.91–14.90) (Fig. 12a).

As per raising mode, the most records were for captive chicken (n = 332), and fewer for free-range chicken (n = 17). The prevalence in chickens of free-range (24.85%, 95% CI: 17.85–31.85) was found to be higher than in captive chickens (7.64%, 95% CI: 7.52–7.76) (Fig. 12b). The prevalence of *S*. Gallinarum varied with economical use of chicken, with the highest prevalence in chicken of dual-purpose (16 records, 25.00%, 95% CI: 16.80–33.19), followed by broiler and layer with the prevalence rate of 20.11% (97 records, 95% CI: 16.80–33.19) and 13.87% (134 records, 95% CI: 13.05–14.69), respectively (Fig. 12c). The millstones of chicken growth stages are different between layer and broiler. Among the four different growth stages of broiler chicken, the prevalence in chicken was extremely high and ranked first (55.34%, 95% CI: 7.32–103.36, 0–14d), followed by teenager (25.33%, 95% CI: 20.41–30.25, 15–30d), adult (23.72%, 95% CI: 17.29–30.16, >31d) and egg (23.03%, 95% CI: 4.49–41.57) (Fig. 12d). For layer chicken, chick possessed the highest prevalence of *S*. Gallinarum as well (18.57%, 95% CI: 7.52–29.61, 1–4w), while the prevalence rates in other growth stages were almost commensurate (adult: 15.66%, 95% CI: 13.57–17.74, >16w; teenager: 15.56%, 95% CI: 12.44–18.68, 5–15w; egg: 15.44%, 95% CI: 6.56–24.32) (Fig. 12e). In hens (13.88%, 95% CI: 12.92–14.83) *S*. Gallinarum was more prevalent than cocks (7.11%, 95% CI: 11.47–13.10) (Fig. 12f).

## Data Records

All data are shared in a single XLSX file with multiple tabs at figshare at the following link (10.6084/m9.figshare.19519402.v1)^32^. Each row corresponds to an individual record, and each column in the sheets represents a variable which is shown as follows:

1. Literature reference: Identifier of references identified for data extraction (The meaning of a blank cell is the same as the one above).
2. of references identified for data extraction (The meaning of a blank cell is the same as the above).
3. Biovar: Biovar of *S*. Gallinarum.
4. Time: Sampling time.
5. Province: Provincial-level information of sampling site.
6. Region: Region-level information of sampling site (Eastern China: Includes Beijing, Tianjin, Hebei, Liaoning, Shanghai, Jiangsu, Zhejiang, Fujiang, Shandong, Guangdong, Hainan, Hong Kong, Macao and Taiwan; Central China: Includes Shanxi, Jilin, Heilongjiang, Anhui, Jiangxi, Henan, Hubei and Hunan; Western China: Includes Chongqing, Sichuan, Yunnan, Guizhou, Tibet, Shaanxi, Gansu, Xinjiang, Qinghai, Ningxia, Guangxi and Inner Mongolia).
7. Country: Country-level information of sampling site.
8. Continent: Continent-level information of sampling site.
9. Source: Reservoir of bacteria.
10. Total sample size: Total number of samples collected.
11. Positive sample size: Number of *S*. Gallinarum positive samples.
12. Positive rate (%): Percentage of *S*. Gallinarum positive samples in total samples.
13. Breed: Chicken breed (classified into foreign and Chinese breeds).
14. Raising mode: The mode of chicken breeding.
15. Economic use: Chicken is sorted by economical purpose.
16. Growth stage: Four growth stages of layer and broiler, respectively.
17. Gender: Chicken sex.

## Technical Validation

The data were all collected from corresponding publications of reliable published sources, with the publication titles and IDs included in the database, if available. Uniform and specific standards were set for the search strategy, publication retrieval and screening and data extraction. To ensure the data were of the highest quality, the authors subsequently performed a rigorous triple check of the data in turn.

## Data Availability

No code was used in this study.
